# Supplementary material for: Translation of immunomodulatory therapy to treat chronic heart failure: Preclinical studies to first in human
Source: PLoS One. 2023 Apr 6;18(4):e0273138. doi: 10.1371/journal.pone.0273138 (PMC10079025; doi:10.1371/journal.pone.0273138)
Supplement: S1 Protocol — (PDF) [file pone.0273138.s002.pdf]

Investigator Initiated Pilot Study to Assess the Safety and Efficacy of a Selective Cytopheretic Device (SCD) to Treat ICU Patients with Acute on Chronic Systolic Heart Failure with Cardiorenal Syndrome Awaiting Left Ventricular Assist Device Implantation

PROTOCOL NUMBER:	HUM00143014
DATE:	NOV 12, 2021
VERSION:	7.0
SPONSOR INVESTIGATOR:	Keith Aaronson, MD, MS
CLINICAL MONITOR:	University of Michigan Institute of Clinical and Health Research (MICHR)<br>Clinical Research Monitoring Program

INVESTIGATOR SIGNATURE PAGE

My signature below certifies that I have read and understand this protocol and agree to conduct the study in accordance with the specified protocol procedures and FDA's Good Clinical Practice requirements.

PRINCIPAL INVESTIGATOR NAME (Printed): ____________________ Date: ____________

PRINCIPAL INVESTIGATOR SIGNATURE: ____________________ Date: ____________

ABBREVIATIONS

ACE	Angiotensin Converting Enzyme
ACDA	Acid Citrate Dextrose-A Solution, USP Formulation
AKI	Acute Kidney Injury
AMI	Acute Myocardial Infarction
ARB	Angiotensin Receptor Blocker
BNP	B-type Natriuretic Peptide
BP	Blood Pressure
BTT	Bridge to Transplant
BUN	Blood Urea Nitrogen
CBC	Complete Blood Count
CD	Cluster or Differentiation
CFR	Code of Federal Regulations
CHF	Chronic Heart Failure
CMP	Comprehensive Metabolic Panel
CO2	Carbon Dioxide
CRRT	Continuous Renal Replacement Therapy
CRF	Case Report Form
CRS	Cardiorenal Syndrome
CVP	Central Venous Pressure
DT	Destination Therapy
ECS	Extracapillary Space
EGFR	Estimated Glomerular Filtration Rate
FDA	Food and Drug Administration
eGFR	Estimated Glomerular Filtration Rate
HF	Heart Failure
HR	Heart Rate
hs-CRP	High Sensitivity C-Reactive Peptide
hs-tnT	High Sensitivity Troponin T
iCa	Ionized Calcium
ICU	Intensive Care Unit
IDE	Investigational Device Exemption
IL	Interleukin
INTERMACS	Interagency Registry for Mechanically Assisted Circulatory Support
IRB	Institutional Review Board
LE	Leukocytes
LVAD	Left Ventricular Assist Device
LVEF	Left Ventricular Ejection Fraction
LVIDd	Left Ventricular Internal Diastolic Diameter
LVGLS	Left Ventricular Global Longitudinal Strain
M	Monocytes
MI	Myocardial Infarction
MØ	Macrophage

MDRD	Modification of Diet in Renal Disease
MR	Mineralocorticoid Receptor
NaCl	Sodium Chloride
NE	Neutrophils
NYHA	New York Heart Association
PA	Pulmonary Artery
PADP	Pulmonary Artery Diastolic Pressure
PAPi	Pulmonary Artery Pulsatility Index
PCWP	Pulmonary Capillary Wedge Pressure
PI	Principal Investigator
Plts	platelet count
RCA	Regional Citrate Anticoagulation
RR	Respiratory Rate
RVF	Right Ventricular Failure
RVFAC	Right Ventricular Fractional Area Change
SAE	Serious Adverse Event
SBP	Systolic Blood Pressure
SCD	Selective Cytopheretic Device
SIRS	Systemic Inflammatory Response Syndrome
sST2	Soluble Suppression of Tumorigenecity-2
TAPSE	Tricuspid Annular Plane Systolic Excursion
Temp	Temperature
TNF-α	Tumor Necrosis Factor-Alpha
WBC	White Blood Count
WRF	Worsening Renal Function

TABLE OF CONTENTS

Abbreviations **3**
Table of Contents **5**
Study Summary **7**
1 Background **13**
1.1 Prior Clinical Experience ***19***
1.2 Rationale for Study and Use of the Selective Cytopheretic Device (SCD) ***20***
2 Product Description **21**
2.1 Intended Use ***21***
2.2 Device Description ***21***
2.3 Device Components ***21***
3 Risk/Benefit Analysis **22**
3.1 Comparison with Other Alternative Devices ***22***
3.2 Minimization of Potential Risks ***22***
3.3 Potential Benefits ***25***
3.4 Justification for the Investigation ***25***
4 Objectives **26**
4.1 Primary Objective ***26***
4.2 Secondary Objectives ***26***
5 Study Outcomes **26**
5.1 Safety Outcomes ***26***
5.2 Efficacy Outcomes ***26***
5.2.1 Principal Outcome **26**
5.2.2 Main Secondary Outcome **27**
5.2.3 Secondary Outcomes **27**
5.2.4 Exploratory Outcomes **27**
6 Study Design **28**
7 Subject Population **28**
7.1 Inclusion Criteria ***28***
7.2 Exclusion Criteria ***29***
8 Study Duration **30**
8.1 Study Duration ***30***
9 Study Procedures **30**
9.1 Recruitment and Screening ***30***
9.1.1 Screening (to be obtained ≤ 2 days prior to enrollment) **30**
9.2 Concomitant Medication ***31***
9.3 SCD Therapy (Days 1-6) ***31***
9.3.1 Description of SCD System **31**
9.3.2 SCD System Perfusion Initiation Protocol **31**
9.3.3 Citrate Anticoagulation Therapy **32**
9.3.4 SCD Prescription Setting **32**
9.3.5 Vascular Access **33**
9.3.6 Sampling **33**

9.3.7 Operating SCD Circuit During Normal Treatment Period **33**
9.3.8 Conclusion of Normal SCD Perfusion **33**
9.3.9 Removal of the SCD **33**
9.3.10 Discontinuation of SCD Therapy Due to Adverse Event **33**
9.3.11 Warnings and Precautions **33**
9.3.12 Required Documentation **34**
9.3.13 Hemodialysis Cartridges **34**
9.3.14 Disconnecting from the SCD Cartridge **34**
9.4 Study Evaluations 34
9.4.1 Each Day of SCD Therapy **34**
9.4.2 LVAD Implantation **36**
9.4.3 Follow-up after LVAD Implantation (7 days and 30 days post implantation (± 1 week)) **36**
9.4.4 Follow up for non LVAD subjects (30 days ± 2 weeks) after last SCD treatment **36**
9.4.5 Schedule of Events Table **37**
9.4.6 Treatment Interruption Criteria **39**
9.4.7 Treatment Discontinuation Criteria **39**
9.4.8 Clinical Trial Termination Criteria **39**
9.5 Device Accountability 40
10 Data Analysis and Statistical Considerations 40
10.1 Sample Size 40
10.2 Screening 40
10.3 Subject Characteristics 40
10.4 Outcome Criteria 40
11 Ethical and Regulatory Considerations 41
11.1 Informed Consent 41
11.2 Institutional Review Board 41
11.3 Complications and Adverse Events 41
11.3.1 Serious and Unanticipated Adverse Device Effects **43**
11.4 Monitoring 43
11.5 Independent Data Review and Data and Safety Monitoring Committee .. 44
11.6 Source Documents / Case Report Forms 44
11.7 Deviation from the Protocol 44
12 References 45

STUDY SUMMARY

**Study Title**	Investigator Initiated Pilot Study To Assess the Safety and Efficacy of a Selective Cytopheretic Device (SCD) to Treat ICU Patients With Acute on Chronic Systolic Heart Failure With Cardiorenal Syndrome Awaiting Left Ventricular Assist Device Implantation
**Study Phase**	Phase 2
**Product Description**	Selective Cytopheretic Device (SCD) is comprised of tubing, connectors and a synthetic membrane cartridge that is connected to a commercially available Renal Replacement pump (a Gambro Prismaflex pump system RRT device). The SCD's synthetic membrane cartridge is a F40 Fresenius hemodialysis cartridge with blood flow directed via side ports. Blood is pumped by the RRT pump through the extracapillary space (ECS) of the SCD. Blood circulates through this space and is returned to the patient via the venous return line of the circuit. Regional citrate anticoagulation is used for the entire SCD blood circuit. When utilized with this flow pathway and in the presence of regional citrate anticoagulation, the SCD membrane binds activated leukocytes and modulates inflammation.
**Rationale**	Individuals hospitalized for acute exacerbations of advanced chronic systolic heart failure are at high risk of death. Mortality risk increases in patients with cardiorenal syndrome (CRS) and acutely worsening renal function or with right ventricular failure, both of which limit the therapies available to patients. Intravenous inotropic therapy is often employed but frequently unsuccessful in these patients.<br><br>LVAD therapy prolongs survival and enhances quality of life in carefully selected patients with chronic systolic heart failure, both when used as a bridge to a heart transplant (BTT) or as permanent or destination therapy (DT). However, severe right ventricular failure is a contraindication to isolated LVAD implantation and severe renal dysfunction (eGFR $\leq$ 30 ml/min/1.73 m^2) is a contraindication to both LVAD therapy and heart transplantation.<br><br>Chronic inflammation promoted by monocyte/macrophage dysregulation has been associated with chronic heart failure (CHF) progression and poor outcome. CHF is now considered a multisystem disease process which involves not only the cardiovascular system but also renal, neuroendocrine and immune systems. Although it is not

* If PCWP cannot be obtained, PADP will be used in its place. When utilizing PADP in place of PCWP for change measures, comparisons will be made to baseline PADP.
** eGFR calculated using the 4-variable MDRD equation
*** Recognizing that this is not a steady state creatinine
**** This will be obtained from the LV tissue removed for placement of the LVAD inflow cannula as part of usual clinical care

	clear what activates the chronic immune system in CHF, the cardiodepressant effects of this activation have been well characterized.<br><br>AKI promotes a systemic inflammatory response syndrome (SIRS) which results in systemic microvascular damage and, if severe, multi-organ dysfunction. Activated circulating leukocytes, especially neutrophils, play a central role in this process as well.<br><br>This proposal plans to evaluate a novel immunomodulatory device, called selective cytopheretic device (SCD), on the immune dysregulated state of CHF with CRS and/or right ventricular failure and to assess the benefit of this innovative strategy to improve cardiovascular and renal function.
**Primary Objective**	To evaluate the safety of 6-hour daily SCD treatments with regional citrate anticoagulation (RCA) in intensive care unit (ICU) patients with NYHA Stage IIIB or IV acute on chronic systolic heart failure (HF),and worsening renal failure (WRF) due to cardiorenal syndrome (CRS) or severe right ventricular failure (RVF), when used as a bridge to durable left ventricular assist device (LVAD) implantation.
**Secondary Objectives**	To evaluate the effect of 6-hour daily SCD treatments with RCA to improve renal function while maintaining or reducing pulmonary capillary wedge pressure (PCWP) in ICU patients with NYHA Stage IIIB or IV acute on chronic systolic HF and WRF due to CRS, when used as a bridge to durable LVAD implantation.
**Safety Outcomes**	1. Need for continuous IV <u>vasopressor</u> support > 5 norepinephrine equivalents > 4 hours following termination of daily SCD therapy session [see text]<br>2. MI<br>3. Death<br>4. Adverse events due to:<br>a. SCD<br>b. Central venous catheter<br>c. RRT device
**Efficacy Outcomes**	*<u>Principal Outcomes</u>*<br>Among subjects with WRF, the percentage of subjects with reversal of WRF (≥ 0.5 mg/dL reduction of serum creatinine from level at study entry), and achieving an eGFR > 30 ml/min/1.73 m2 <u>and</u> PCW* at or below level at study entry at termination of SCD therapy

* If PCWP cannot be obtained, PADP will be used in its place. When utilizing PADP in place of PCWP for change measures, comparisons will be made to baseline PADP.
** eGFR calculated using the 4-variable MDRD equation
*** Recognizing that this is not a steady state creatinine
**** This will be obtained from the LV tissue removed for placement of the LVAD inflow cannula as part of usual clinical care

	Among subjects with severe right ventricular failure, the percentage of subjects who no longer have severe right ventricular failure, as evidenced by absence of 3 or more of the following 4 indicators of right ventricular failure:<br>1) CVP > 16 mmHg<br>2) CVP/PCWP > 0.65<br>3) RVSWI < 300 mmHg * ml/m<br>4) PAPi < 2<br><br>*Main Secondary Outcome*<br>Percentage of subjects receiving a left ventricular assist device<br><br>*Secondary Outcomes* (unless otherwise noted, each assessed as change from onset of intervention to 3 and 6 days after initiation of SCD treatments and from onset of intervention to end of SCD support prior to LVAD implantation)<br>Laboratory Outcomes<br><br>1. Change in 24 hour urine volume and sodium, creatinine, urea, creatinine clearance and urea clearance<br>2. Change in PCWP*<br>3. Change in serum sodium, potassium, dissolved CO2, BUN, creatinine<br>4. Change in RV fractional area change, TAPSE and RV global longitudinal strain<br><br>Clinical Outcomes<br>1. Percentage of subjects with ≥ 0.5 mg/dL reduction of serum creatinine from level at study entry, and PCWP* ≤ 18 mmHg)<br>2. Percentage of subjects receiving a left ventricular assist device with serum creatinine ≥ 0.5 mg/dL below level at study entry 30 days following discontinuation of SCD<br><br>*Exploratory Outcomes* (unless otherwise noted, each assessed as change from onset of intervention to 3 and 6 days after initiation of SCD treatments and from onset of intervention to end of SCD support prior to LVAD implantation)<br>1. Change in cystatin-C, BNP, hs-tnT, IL-6, TNF-a, IL-1β, hs-CRP, IL-10, norepinephrine, renin, aldosterone, neopterin, osteopontin, sST2.

* If PCWP cannot be obtained, PADP will be used in its place. When utilizing PADP in place of PCWP for change measures, comparisons will be made to baseline PADP.
** eGFR calculated using the 4-variable MDRD equation
*** Recognizing that this is not a steady state creatinine
**** This will be obtained from the LV tissue removed for placement of the LVAD inflow cannula as part of usual clinical care

	2. Change in circulating leukocyte phenotypes (neutrophils, monocytes) with phenotypic cell sorting<br>3. Characterization of macrophage subpopulations within the left ventricular myocardium obtained at the time of LVAD implantation****
**Assessment of Device Integrity and Performance**	Criteria for assessment of device integrity and performance include:<br>1. Significant clotting within the device as assessed by visual inspection.<br>2. Evidence of leakage (i.e., cracking/breakage of a port, connector, hemofilter cartridge or tubing).<br>3. Any unforeseen malfunction that results in the need for discontinuation.
**Study Populations**	Adult patients in an ICU with NYHA class IIIB or IV chronic systolic heart failure will be screened daily and identified for participation in the trial
**Study Design**	Single arm, open label, uncontrolled, before-after, pilot study
**Number of Subjects**	Ten (10) patients will be enrolled in this study
**Number of Centers**	There will be 1 clinical center, the University of Michigan Hospital
**Duration of Subject Participation**	Each subject will be followed for 30 days following discontinuation of SCD therapy
**Inclusion Criteria**	1) Primary hospitalization for acute decompensated chronic systolic heart failure<br>2) Potential LVAD candidate with:<br>a) Left ventricular ejection fraction (LVEF) ≤25% (for potential destination therapy) or ≤ 35% (for potential bridge to transplantation) as confirmed by baseline imaging procedure<br>b) NYHA class IIIB or IV chronic (≤ 90 days) systolic heart failure, with failure to respond to optimal medical therapy (beta blocker, ACE inhibitor or ARB or valsartan/sacubitril, aldosterone antagonist, unless not tolerated or contraindicated, and loop diuretic, as needed) for 45 of the last 60 days<br>c) Known previous peak exercise oxygen consumption < 14 mL/Kg/min or if unable to exercise, dependent on an intra-aortic balloon pump, short-term mechanical circulatory support device or intravenous inotropes unless inotropes contraindicated for clinical reasons (e.g., ventricular arrhythmias)

* If PCWP cannot be obtained, PADP will be used in its place. When utilizing PADP in place of PCWP for change measures, comparisons will be made to baseline PADP.
** eGFR calculated using the 4-variable MDRD equation
*** Recognizing that this is not a steady state creatinine
**** This will be obtained from the LV tissue removed for placement of the LVAD inflow cannula as part of usual clinical care

	3) Baseline eGFR** ≥ 40 ml/min/1.73 m^2 (baseline defined as the highest known eGFR within 90 days of study enrollment)<br>4) One or both of the following two criteria:<br>a) Worsening renal failure (WRF), defined for the purposes of this study as<br>1) Increase serum creatinine ≥ 0.5 mg/dL from baseline (baseline defined as the lowest known serum creatinine within 90 days of study enrollment)<br>***AND***<br>2) eGFR** ≤ 30 ml/min/1.73 m^2 based on serum creatinine at enrollment**<br>*AND*<br>3) Cardiorenal syndrome is the most likely explanation for WRF<br>*AND*<br>4) Intolerant or inadequately responsive to standard of care diuretic therapy<br>b) Severe right ventricular failure (RVF), defined as meeting at least 2 of the following 4 criteria<br>1) Central venous pressure > 16 mmHg<br>2) Central venous pressure/Pulmonary wedge pressure >0.65<br>3) Right ventricular stroke work index < 300 mmHg * ml/m^2<br>4) Pulmonary artery pulsatility index (PAPi) < 2,<br>5) PA catheter in place at the time of enrollment<br>6) PCW ≥ 20 mmHg<br>7) Age ≥ 21 and ≤ 75 years
**Exclusion Criteria**	1. Any clear contraindication to LVAD therapy that is unlikely to resolve with improvement in renal function, right ventricular function and volume status<br>2. Prior sensitivity to dialysis device components<br>3. Bacteremia<br>4. Temperature ≥ 101.5 F or WBC ≥ 10,000 K/uL or any patient with suspected systemic infection.<br>5. Active malignancy requiring chemotherapy, biological therapy or radiation therapy<br>6. The use of intravenous iodinated contrast agent within the prior 72 hours or the anticipated use of such an agent during SCD therapy<br>7. Need for intravenous vasopressor (i.e., phenylephrine, vasopressin), intravenous vasoconstricting inotrope (i.e., norepinephrine or epinephrine) or dopamine > 3 mcg/kg/min. *(Note: use of vasodilating inotropes [i.e., dobutamine and*

* If PCWP cannot be obtained, PADP will be used in its place. When utilizing PADP in place of PCWP for change measures, comparisons will be made to baseline PADP.
** eGFR calculated using the 4-variable MDRD equation
*** Recognizing that this is not a steady state creatinine
**** This will be obtained from the LV tissue removed for placement of the LVAD inflow cannula as part of usual clinical care

	milrinone] or dopamine at ≤ 3 mcg/kg/min will not preclude study inclusion)<br>8. Persistent SBP < 80 mmHg<br>9. WBC < 4000 K/uL<br>10. Platelets < 100,000 K/uL<br>11. Serum creatinine > 4 mg/dL or receiving dialysis / CRRT<br>12. Acute coronary syndrome within the past month<br>13. Women who are pregnant, breastfeeding a child, or trying to become pregnant.<br>14. Subject not able to sign informed consent, unless they have a legally authorized representative (LAR)<br>15. Concurrent enrollment in another interventional clinical trial. Patients enrolled in clinical studies where only measurements and/or samples are taken (i.e., no test device or test drug used) are allowed to participate<br>16. Use of any other investigational drug or device within the previous 30 days
**Intervention**	1. Phase 2, single arm, open-label, uncontrolled, before-after study<br>2. Experimental intervention: Selective Cytopheretic Device (SCD, an FDA cleared F40 Fresenius hemodialysis cartridge with blood flow directed via side ports.)<br>3. Subjects will be placed on SCD for planned 6 hours of daily therapy for up to 6 consecutive days.<br>4. At or before 6 days of SCD therapy, a decision will be reached by the patient's clinicians as to whether or not the subject is eligible for LVAD surgery.<br>5. The option to continue SCD therapy will terminate if it is determined that the subject is not a candidate for LVAD therapy.

* If PCWP cannot be obtained, PADP will be used in its place. When utilizing PADP in place of PCWP for change measures, comparisons will be made to baseline PADP.
** eGFR calculated using the 4-variable MDRD equation
*** Recognizing that this is not a steady state creatinine
**** This will be obtained from the LV tissue removed for placement of the LVAD inflow cannula as part of usual clinical care

1 BACKGROUND

Without heart transplantation or mechanical circulatory support, hospitalized individuals with Stage D acute on chronic systolic heart failure have a life expectancy of days to weeks. Their prognosis is especially bleak when their heart failure is complicated by cardiorenal syndrome (CRS), a pathophysiologic disorder of the heart and kidneys whereby acute or chronic dysfunction of one organ may induce acute or chronic dysfunction of the other [1]. An acute cardiac insult resulting in a substantial impairment of cardiac function, such as myocarditis or a large myocardial infarction, may result in acute renal dysfunction (Type I CRS). Chronic heart failure is associated with chronic renal dysfunction (Type II CRS) in 32% of patients[2]. While elevated central venous pressure, a manifestation of right ventricular failure (see below) appears to be associated with CRS, in at least some studies of patients with advanced systolic heart failure, there has been no association between cardiac index and CRS.[3,4] Renal hypoperfusion, neurohormonal activation, nephrotoxic effects of heart failure medications likely contribute to the syndrome.

Mortality risk among hospitalized heart failure patients is inversely related to renal function at the time of admission, with a 23% higher mortality for every 10 ml/min/1.73 m^2 lower estimated glomerular filtration rate (eGFR)[5]. Hospitalization for heart failure results in acute worsening of renal function (WRF), usually defined as ≥0.3 mg/dL rise in serum creatinine, in 23% of patients[2], with progressively increased risk with greater baseline renal dysfunction[6]. Precipitants of WRF include an additional decline in cardiac output, an increase or decrease in intravascular volume or central venous pressure, hypotension and nephrotoxic drugs, but an acute precipitant cannot be identified in all patients. Worsening renal function in patients with heart failure is associated with nearly a doubling of mortality risk.[2] In another study, heart failure patients who experienced WRF (≥ 0.3 mg/dL) and remained congested at the time of discharge were more than 5 times more likely to die or receive an urgent heart transplantation in the following year.[7]

Optimization of cardiac hemodynamics, through indwelling pulmonary catheter monitoring with diuretic manipulations and intravenous inotropic agents, may result in temporary improvement in renal function. However, further activation of the renin-angiotensin-aldosterone and sympathetic nervous systems, and induction of supraventricular or ventricular arrhythmias may precipitate worsening renal function, progression of heart failure and death. Neither intraaortic balloon pumps nor other temporary forms of mechanical circulatory support reliably improve kidney function in this setting. For refractory patients, LVAD implantation is the only remaining treatment option. However, left ventricular assist device (LVAD) implantation in the setting of renal dysfunction is associated with substantially higher early and midterm mortality in the INTERMACS registry.[8]. Of 4917 patients who received a continuous flow LVAD in this analysis, only 6% had an estimated creatinine clearance ≤ 30 ml/min (i.e., severe renal dysfunction) and their mortality was 22% at 3 months. As such, patients with eGFR ≤ 30 ml/min/ 1.73 m^2 are excluded from LVAD candidacy at most centers, including our own.

Individuals with severe chronic left ventricular systolic heart failure may also be affected by right ventricular systolic heart failure (RVF). RVF may directly result from the same underlying disease process that caused left ventricular failure, or may be a consequence of the hemodynamic derangements of left ventricular failure of any etiology. LVAD therapy addresses left ventricular systolic failure but does not address and may even worsen RVF. Early RVF is the major cause of death in the early postoperative period. LVAD candidates routinely undergo preoperative hemodynamic assessment for indicators of RVF. Patients found to be at high risk for RVF (e.g., high CVP, high CVP/PCWP, low RVSWI, low PAPi) are not offered LVAD therapy. Thus there is an *unmet need for a therapy that can improve heart and kidney function in cardiorenal syndrome* and Stage D heart failure to allow LVAD implantation when standard therapies have failed to do so.

Innovative Therapy

Strategies to reduce the cardiodepressant effects of inflammation rather than enhancing myocardial contractility have only recently been considered. Although chronic inflammation promoted by monocyte/macrophage dysregulation has been associated with CHF progression and poor outcome, an approach to immunomodulate the dysregulated leukocyte activity in CHF is currently an unrecognized and untested paradigm. CHF is now considered a multisystem disease process which involves not only the cardiovascular system but also renal, neuroendocrine and immune systems. Although it is not clear what activates the chronic immune system in CHF, the cardiodepressant effects of this activation have been well characterized. This proposal plans to evaluate a novel immunomodulatory device, called selective cytopheretic device (SCD), on the immune dysregulated state of CHF and to assess the benefit of this innovative strategy to improve the cardiovascular and renal function in CRS.

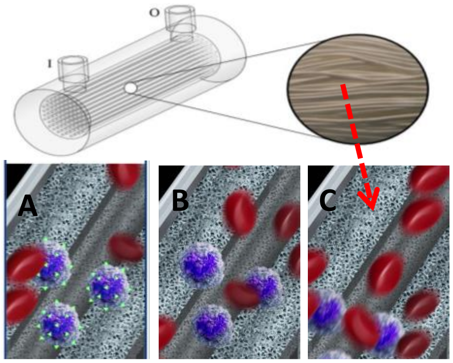

Figure 1. SCD Design Schematic and Potential Mechanism of Action Diagram.

SCD consists of a polycarbonate housing with an inlet (I) and outlet (O) for blood, and potted polysulfone-based fibers. Circular blow-up of the fibers are shown, with dashed red arrow pointing to panel C, showing release of immuno-modulated leukocytes (LE), Panel A: binding (catch) of activated LE, Panel B: bound LE, in the SCD low Ca environment, are deactivated. Erythrocytes are red, LE are purple and LE binding sites, acutely mobilized to the surface of activated LE are green (Panel A).

Dr. H. David Humes, Professor of Internal Medicine, and his group have developed a biomimetic membrane cell processing device, named the Selective Cytopheretic Device (Figure 1). This developing commercial product licensed to CytoPherx, Inc. (a University of Michigan spinoff) is identical to hemodialysis cartridges (F series) manufactured by Fresenius with blood flow directed via side ports. End ports remain capped as they are received from the

manufacturer with no flow. The device itself is not altered, but the blood flow through the device will be altered because of the tubing connected to the side ports rather than the end ports.

This device, when incorporated into an extracorporeal blood circuit, preferentially binds activated leukocytes (LE), and in the low calcium environment afforded by regional citrate anticoagulation (RCA), the bound LE are deactivated and released back to the systemic circulation. This device is identical to a polysulphone membrane dialyzer but with the blood flow path directed to the outside of the hollow fiber membrane rather than the inside of the membrane. The blood flow path results in low shear forces similar to capillary shear so the membrane has selectivity to bind activated LE. This continuous cell processing activity results in measurable diminution of excessive inflammatory responses in a variety of preclinical and clinical disorders.

Inflammation and Myocardial Injury. A vigorous inflammatory response occurs immediately after reperfusion to the ischemic myocardium since various molecular signals are generated by injured endothelial cells and cardiomyocytes. This response is eventually important in the wound healing and remodeling necessary to reestablish cardiac performance but is excessive and maladaptive. The increase in circulating levels of innate immune cells, including neutrophils (NE) and monocytes (M), arise both from the splenic M reservoir pool and the bone marrow precursor pool [9] to produce the initial pro-inflammatory response.

The magnitude of NE infiltration into the damaged area of the heart accentuates the degree of injury and cardiac dysfunction. Accordingly, NE depleting strategies have been demonstrated to reduce infarct size [10,11]. The role of the circulating M and its transformation into a tissue Mϕ is now acknowledged to be central to the injury and repair phases of this injury process. After myocardial injury the initial inflammatory response consists of recruitment of activated NE and M within the damaged area of the heart [12,13]. The M/Mϕ subset that dominates in this early phase, peaking at 72h, is predominately a classical M1 pro-inflammatory phenotype ($CD14^{++}$ $CD16^{-}$ in humans, $Ly6C^{+}$ in mice). These M/Mϕ promote degradative processes and phagocytic activity to remove necrotic tissue. A non-classical alternative M2 M/Mϕ phenotype ($CD14^{+}$ $CD16^{+}$ in humans, $Ly6C^{-}$ in mice) emerges early and expands to orchestrate tissue repair [9,12-15]. A balanced M/Mϕ response, both in phenotype and timing, is necessary for optimal repair and healing [9,13]. A number of studies have shown that suppression of the early phase of M1 release from spleen by either angiotensin II blockade or splenectomy reduces infarct size and myocardial dysfunction acutely and sub-acutely [16]. This temporal biphasic M/Mϕ response and the splenic M reservoir have been confirmed in myocardial and splenic tissues in patients with acute myocardial infarction [17]. In addition, more vigorous response of circulating M M1 phenotype after AMI has been correlated to a greater decline in left ventricular ejection fraction 6 mos. after AMI [18]. These longer term effects of the initial M response after ischemic damage to the heart have a critical role in the persistent inflammation and progressive myocardial dysfunction occurring in CHF. The cardiodepressant effects of this immunologic activation have been well characterized [19-23]. Chronic inflammation promoted by M/Mϕ dysregulation has been correlated with CHF progression and poor outcome. The mechanism of mineralocorticoid receptor antagonists to

retard the progression and mortality in CHF patients relates to the ability to block MR activation of cardiac tissue Mϕ to release pro-inflammatory molecules [23,24]. Recent data have demonstrated that activation of M/Mϕ is central to the progressive cardiac fibrosis in CHF and that the spleen is central to the persistent inflammatory state in CHF. The spleen, due to a heightened antigen processing state, promotes splenic M to be released. In a murine model of CHF, these cells home specifically to the myocardium with subsequent infiltration and continuing injury and remodeling [25]. Splenectomy reversed this progressive myocardial damage. Splenic M retain this ability to home and injure myocardial tissue and upon adoptive transfer to a normal mouse promotes immune-mediated progressive cardiac dysfunction [25]. The modulation of this persistent inflammatory state associated with acute and chronic cardiac injury may be an innovative approach to delay progressive CHF**.**

Preliminary Study Results.
Preclinical Studies in Sepsis and AKI: The preclinical data in a 30-35kg pig model of septic shock associated acute kidney injury with SCD therapy have been detailed in the referenced publication [26]. This study demonstrated that SCD therapy had a significant ameliorative effect on sepsis induced organ dysfunction, including cardiac, pulmonary and renal parameters along with an immunomodulatory effect on circulating LE.

SCD therapy is proving to be a platform technology to treat acute and chronic organ dysfunction. In preclinical models, SCD therapy has shown efficacy in acute multiorgan injury in a canine model of chronic heart failure (CHF). SCD therapy also prevents the inflammatory response to prolonged cardiopulmonary bypass, intracerebral hemorrhage (ICH), ischemia/reperfusion injury (IRI), and improves myocardial contractility in CHF. SCD therapy has been tested in 4 clinical studies on 124 patients with positive clinical outcomes and no device related adverse events. A phase II IDE safety and early efficacy trial in pediatric ICU patients with acute renal failure (ARF) and multiorgan dysfunction (MOD) began in September 2016. An FDA/IDE pivotal trial is planned to begin in 2018 in adult ICU patients with ARF and MOD.

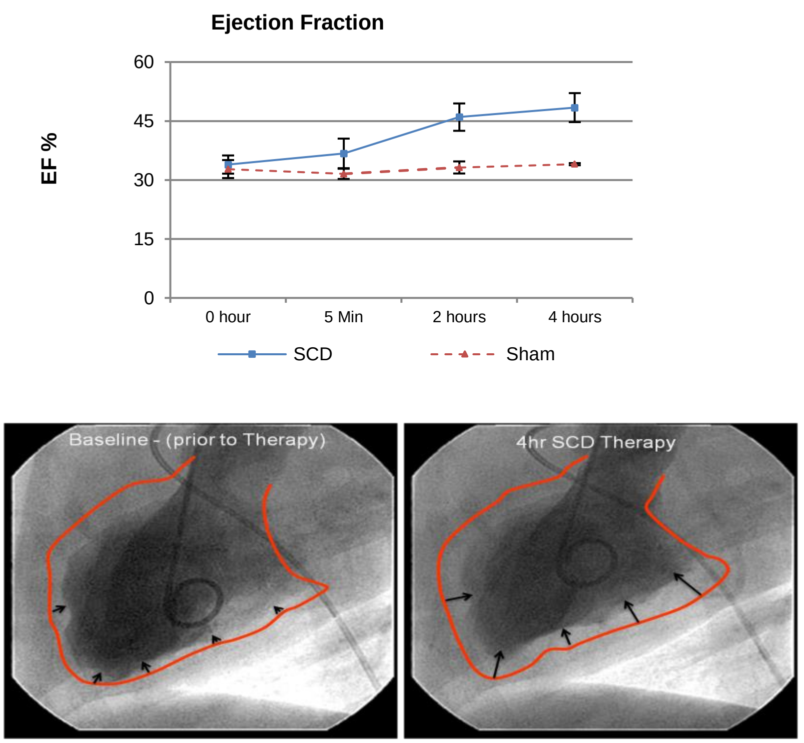

Figure 2. CHF dogs treated for 4h with SCD (n=3) or sham (n=2) therapy.

LVEF (Top Panel) was returned near normal levels of 50-55% under SCD therapy. No effect on EF with sham therapy was observed. Ventriculograms of a CHF dog heart (Bottom Panel) are shown at baseline (before therapy) and at the end of the 4h therapy session. The red line depicts the border of the diastolic silhouette overlayed on the systolic image, demonstrating improved contractility (black arrows) of the left ventricle after SCD therapy.

Acute and Chronic Heart Failure: Inflammation is central to the development of a variety of acute organ failures, including heart, kidney, lung and brain as well as chronic organ dysfunction, including heart, kidney and Type 2 diabetes. Chronic heart failure and acute exacerbations of chronic HF have been increasingly recognized as associated with chronic systemic inflammation [19]. M and tissue Mϕ have been identified as critical sources of systemic

inflammation in CHF [20,31] and cause a decrease in cardiac myocyte contractility [22,31]. The effects of SCD therapy has been evaluated in a preclinical canine model of chronic heart failure developed and fully characterized by Dr. Hani Sabbah at Henry Ford Hospital [32]. The model manifests many of the sequelae of CHF in humans including profound systolic dysfunction, increased systemic vascular resistance and decreased cardiac output. It has been used to predict efficacy of new therapies for treatment of CHF and has provided critical insights into how successful therapeutic interventions reverse maladaptive molecular mechanisms underlying heart failure pathophysiology [33,34]. Five dogs with advanced CHF were used in the study set. The effect of SCD therapy on LVEF in this model (Figure 2) demonstrates that SCD therapy converts viable but non-contracting myocardium to contracting myocardium. The renal effects were also substantive with doubling of urine sodium excretion with SCD therapy compared to baseline. With respect to safety profile, no episodes of arrhythmias or hypotension were observed during the treatment period. Recent data have demonstrated that this SCD effect to improve myocardial contractibility in this canine model persists up to 4 weeks. This improvement correlates to SCD related immunomodulatory effects and diminution in myocardial monocyte/macrophage entry.

Further studies using this canine model were undertaken with funds from NIH grant R43HL118792. Twelve dogs with advanced CHF (EF<35%) were used as follows: 5 control dogs received sham Rx manipulations and 7 dogs received SCD Rx sessions. The sham and SCD Rx sessions were for 4- 6 hours and administered every 48-72 hours for a total of 3 times over a 1-week period. At various study points, hemodynamic and ventricular function parameters were measured and blood samples for the assessment of various biologic parameters were taken. At the 4wk study end-point, heart and spleen were harvested, and peritoneal lavage performed to obtain Mϕ, in order to assess SCD Rx impact on LE inflammatory parameters. Additionally, LE that had bound to the SCD during the 4-6-hour Rx period were eluted, enumerated with population differentials and assessed for LE activation levels.
SCD Rx enhanced overall cardiac function, as indicated by increased LVEF% that was sustained over the 4-week follow-up period (Figure 3). SCD Rx also demonstrated enhancement of other cardiac parameters when comparing wk 0 to post Rx: End-systolic volume (ESV) had an improvement of 10.5±0.8%, cardiac output (CO) 30.6±7.8%, stroke volume (SV) 24.2±4.0% and left ventricular end-diastolic pressure (LVEDP) 7.7±2.3%. Of importance, EF, ESV, CO, SV and LVEDP remained unchanged in the sham group. Left ventriculograms also were shown to convert viable, but **non-contracting** myocardium, to **contracting** myocardium, similar to Figure 2. With respect to safety profile, SCD Rx elicited no episodes of arrhythmias or hypotension.

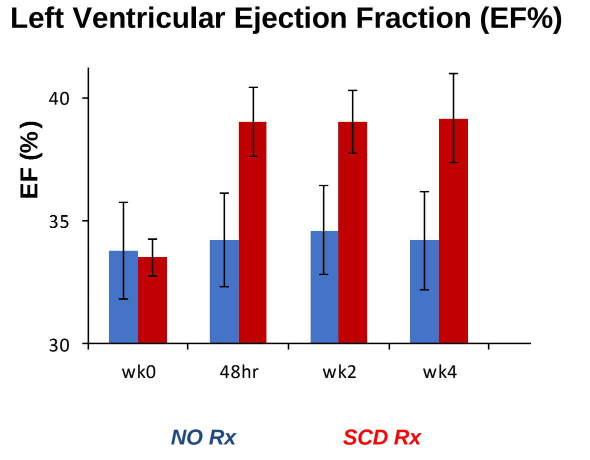

Figure 3. SCD impact on ejection fraction (EF). EF was measured at wk0 (pre-Rx) and over the 4-week study time course for all CHF dogs. SCD Rx dogs displayed a sustained EF improvement over the 4-week follow-up having a significant improvement over wk0 (p<0.05). The No Rx cohort had no change in EF over the 4-week period.

Systemic BNP, NE elastase, TNFα and IL-6 were all lower after SCD Rx. NE and M activation levels declined after SCD Rx. Resident Mϕ density in cardiac tissue was lower in SCD treated dogs. The increased cell count/gm tissue associated with spleen under the CHF state was returned to near normal levels at 4 weeks post-SCD Rx. The elevated peritoneal Mϕ number associated with CHF was also lower after SCD Rx, with Mϕ phenotype preference moved from the pro-inflammatory M1, seen in the No Rx dogs, to the reparative/anti-inflammatory M2 phenotype seen in the SCD Rx dogs. This latter data suggest that SCD therapy has an effect to alter systemic Mϕ phenotype.

All of these outcomes suggest SCD therapy over a 1-week course improves cardiovascular parameters associated with sustained less proinflammatory profiles systemically for greater than 4 weeks post treatment.

1.1 Prior Clinical Experience

The clinical evaluation of SCD therapy has been completed in 3 clinical trials in ICU adult patients with AKI requiring continuous renal replacement therapy (CRRT) and multi organ dysfunction (MOD) (IDE G090189- IDE held by CytoPherx). These trials have demonstrated an excellent safety profile and suggested efficacy impact [27-30]. Leukopenia and sustained thrombocytopenia were not observed; accelerated renal recovery with RRT discontinuation and an approximately 15-20 percent or greater improvement in survival rates has been observed compared to conventional RRT. This device is added in series in the CRRT circuit after the standard HD filter. Of note, in all 3 trials SCD therapy was well tolerated, without significant effects on hematological parameters, including white blood cell and platelet counts, and with no unanticipated serious adverse events related to SCD therapy.

Due to these favorable results in 3 trials, across 19 different clinical sites and 56 patients, a Phase III controlled, randomized, multicenter clinical trial was conducted. [30] The control group received standard CRRT with regional citrate anticoagulation (RCA) while the treated group received CRRT with RCA plus up to 7 days of SCD therapy. No significant difference in 60-day mortality was observed between the treated (27/69; 39%) and control patients (21/59; 36%, with six patients lost to follow up) in the intention to treat (ITT) analysis. However, due to a national shortage of calcium, there was a tendency for investigators to minimize citrate infusion rates, resulting in intra-circuit ionized calcium levels (iCa) above the protocol recommended range of 0.25 to 0.40 mmol/L in the majority of subjects. Therefore, the study was stopped prematurely by the sponsor, and an analysis was performed on a modified data from those patients who maintained a post-filter iCa level ≤ 0.4 mmol/L for ≥ 90% of the therapy time. Of the 19 SCD subjects (CRRT+SCD) and 31 control subjects (CRRT alone) who met this criterion, 60-day mortality was 16% (3/ 19) in the SCD group compared to 41% (11/27) in the CRRT alone group (p = 0.11). Dialysis dependency showed a borderline statistically significant difference between the SCD treated versus control CRRT alone patients maintained for ≥ 90% of the treatment in the protocol's recommended (r) iCa target range of ≤ 0.4 mmol/L with values of 0% (0/16) and 25% (4/16), respectively (P = 0.10). When the riCa treated and control subgroups were compared for a composite index of 60-day mortality and dialysis dependency, the percentage of SCD treated

subjects was 16% versus 58% in the control subjects (p<0.01). The incidence of serious adverse events (SAEs) did not differ between the treated (45/69; 65%) and control groups (40/65; 63%; p = 0 86) and there were no SAEs classified as "definitely" device related.
This analysis clearly demonstrates a clinical effectiveness endpoint without safety issues and provides substantive support to move this technology to other patient populations. A similar pediatric clinical trial (NCT 02820350l) is ongoing with funding from the FDA Office of Orphan Products Development and is demonstrating similar safety and efficacy results.

1.1.1 First in Man
One patient in the adult AKI Phase III trial with severe CRS (EF 20%) with acute tubular necrosis was enrolled and randomized to the SCD treatment arm. This patient was 15 kg over his dry weight with a cardiac index of 1.4, pulmonary capillary wedge pressure of 30 mmHg, BUN 64 and serum creatinine of 3.38 mg/dl. His urine output and net volume removal were resistant to intravenous loop diuretics, dobutamine, and milrinone. SCD therapy was initiated with CRRT and he demonstrated an acute and sustained doubling of his urine output, and the ability to sustain ultrafiltration without hypotension to achieve a 15 kg net volume removal over 7 days. This serendipitous observation supports the preclinical animal data and provides first in man observations in CRS

1.2 Rationale for Study and Use of the Selective Cytopheretic Device (SCD)

Individuals hospitalized for acute exacerbations of advanced chronic systolic heart failure are at high risk of death. Mortality risk increases in patients with CRS and acutely worsening renal function. Intravenous inotropic therapy is often employed but frequently unsuccessful in these patients.

LVAD therapy prolongs survival and enhances quality of life in carefully selected patients with chronic systolic heart failure, both when used as a bridge to a heart transplant (BTT) or as permanent or destination therapy (DT). However, severe renal dysfunction (eGFR ≤ 30 ml/min/1.73 m2) and severe right ventricular failure are both considered a contraindication to LVAD therapy and severe renal dysfunction (eGFR ≤ 30 ml/min/1.73 m2) remains a contraindication to heart transplantation.

Chronic inflammation promoted by monocyte/macrophage dysregulation has been associated with chronic heart failure (CHF) progression and poor outcome. CHF is now considered a multisystem disease process which involves not only the cardiovascular system but also renal, neuroendocrine and immune systems. Although it is not clear what activates the chronic immune system in CHF, the cardiodepressant effects of this activation have been well characterized.

AKI promotes a systemic inflammatory response syndrome (SIRS) which results in systemic microvascular damage and, if severe, multi-organ dysfunction.[35,36] Activated circulating leukocytes, especially neutrophils, play a central role in this process as well.[37]

This proposal plans to evaluate a novel immunomodulatory device, called selective cytopheretic device (SCD), on the immune dysregulated state of CHF with CRS and to assess the benefit of this innovative strategy to improve cardiovascular and renal function.

2 PRODUCT DESCRIPTION

2.1 Intended Use

The SCD is being investigated to treat ICU patients with acute on chronic systolic heart failure and worsening renal function due to cardiorenal syndrome while awaiting LVAD implantation.

2.2 Device Description

.

The SCD is comprised of a dialyzer, tubing, connectors and a synthetic membrane cartridge that is connected to a commercially available Renal Replacement pump (a Gambro Prismaflex ® RRT device).

2.3 Device Components

The SCD System is composed of the following devices:

- Hemodialysis machine: Gambro Prismaflex System 7.10: FDA Cleared 510(k) K131516
- Commercial dialysis tubing set: Prismaflex System HF1000 dialysis tubing set: FDA Cleared 510(k) K042938
- Hemodialyzer: Prismaflex System HF1000 hemofilter set: FDA Cleared 510(k) K042938
- SCD blood tubing set: Medtronic Intersept Custom Tubing Pack. FDA Cleared 510(k) K800178
- SCD-F40: Fresenius polysulfone hemofilter (F-40) FDA Cleared 510(k) K921134

The SCD-F40 is a single use hollow fiber synthetic membrane unaltered cleared F40S hemodialyzer cartridge from Fresenius with blood flow directed via side ports. End ports remain capped as they are received from the manufacturer with no flow. The device itself is not altered, but the blood flow through the device will be altered because of the tubing connected to the side ports rather than the end ports. The blood flow through this cartridge is designed to selectively sequester and release activated neutrophils and monocytes resulting in an immunomodulatory effect of circulating leukocytes.

The two cartridges (Prismaflex System HF1000 hemofilter and the Fresenius F-40 hemofilter) are connected in series to a commercially available FDA cleared hemodialysis device (Gambro Prismaflex System 7.10). Blood from the hemodialysis circuit is diverted after the dialyzer through the extra capillary space (ECS) of the SCD-F40 using the SCD blood tubing set. Blood circulates through this space and it is returned to the patient via the venous return line of the hemodialysis circuit. Regional citrate anticoagulation is used for the entire hemodialysis and SCD-F40 blood circuits. The extracorporeal perfusion circuit uses standard dialysis arteriovenous blood tubing and is schematized in the Figure 4 with the hemodialysis dialyzer and the SCD-F40 in series.

The first dialysis cartridge is Prismaflex System HF1000 hemofilter and the second cartridge is the SCD-F40 (Fresenius polysulfone hemofilter (F-40) with the tubing connected to the side ports rather than the end ports). The SCD Blood Tubing Set blood lines maintain a pressure fit over the side ports of the SCD device in the same manner perfusion blood tubing is connected to a hemoconcentrator.

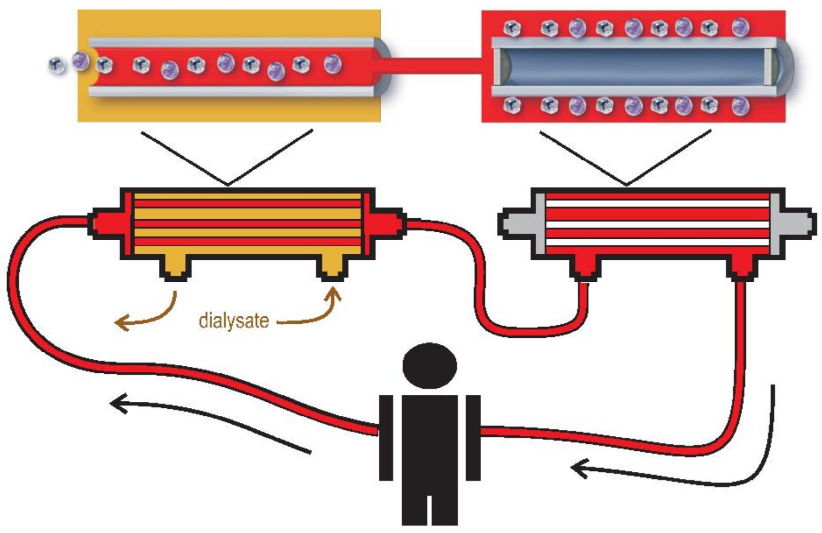

Figure 4. SCD Device Schematic.

3 RISK/BENEFIT ANALYSIS

3.1 Comparison with Other Alternative Devices

There are no presently approved devices for the treatment of cardiorenal syndrome.

3.2 Minimization of Potential Risks

Clinical Risk Factors and Manner of Minimization:

Neutropenia

The precise mechanism of action of the SCD appears to be an immune-modulatory process that suppresses leukocyte activation, a trigger of systemic inflammatory response syndrome (SIRS) and multi-system organ failure. The modulation of the proinflammatory state, mostly due to a monocyte effect, also allows recovery of renal function in ARF and is postulated to be responsible for the improvement in cardiac function in animal models of heart failure.

The citrate-treated SCD acts as a selective cytopheretic device to sequester and inhibit potentially damaging circulating leukocytes. Since the SCD sequesters WBC's, neutropenia is a risk.

Risk Minimization
The risk of neutropenia is mitigated by performing and monitoring complete blood counts including WBC and differential frequently during SCD therapy. Consideration of discontinuing SCD therapy if a critical value of <500 neutrophils/cm^3 occurs based upon PI judgment.

Human Experience
In 140 patients [15 ESRD and 125 AKI] who have been treated with the SCD, no neutropenia AE's have been reported.

Blood Clotting in the RRT/SCD Circuit
Blood clotting is a known occurrence with conventional RRT. There is no reason to believe that blood clotting is more likely with the SCD device in the extracorporeal circuit. However, if this occurs, a change in the extracorporeal circuit and tubing results in a possible loss of 100 cc of blood. Blood transfusions may be necessary to replete the patient's blood volume (see below under Hypotension).

Risk Minimization
Michigan Medicine has extensive experience and a well-established regional citrate anticoagulation protocol. Our study site currently uses a citrate anticoagulation protocol in CRRT patients and has safety monitoring of ionized calcium.

Human Experience
Prior human experience exists related to clotting within the dialysis circuit and/or the SCD cartridge. All reported circuit clotting events were deemed to arise in the circuit prior to the SCD and not within the SCD.

In the 15 patient ESRD study of the SCD device at Henry Ford Hospital (previously conducted Non-Significant Risk (NSR) Trial -NSR as determined by the local Institutional Review Board) only one episode of clotting occurred at the "header" of the cartridge and did not require changing the cartridge. The pediatric population study of the SCD device conducted under the CytoPherx held IDE G150179 also utilized the Gambro Prismaflex dialysis system with flow rates of 50-150mL/min, and there were no incidences of clotting within the system.

Failure to Maintain Blood Circuit Ionized Calcium (iCa) below 0.40 mmol/L
Clinical data demonstrates that SCD therapy requires the extracorporeal blood circuit ionized calcium (iCa) to be consistently below 0.40 mmol/L to be clinically efficacious. If iCa values are not below 0.40 mmol/L for 90% of treatment time, SCD therapy loses its

clinical efficacy on mortality and dialysis dependency.

Risk Minimization
Blood circuit post-filter iCa levels will be measured every two hours during each SCD therapy session to ensure that it is below 0.40 mmol/L.

Human Experience
In all clinical studies to date, SCD therapy was efficacious when post-filter blood circuit iCa was below 0.40 mmol/L for 90% of measured values. This efficacy on mortality and dialysis dependency at 60 days was not seen when iCa was not maintained at ≥0.40 mmol/L for 90% of treatment time.

Metabolic Derangements

a. *Alkalosis*: With the 6 hour citrate infusion substantive alkalemia (pH>7.55) secondary to citrate metabolism will not occur. If the patient's arterial pH increases to >7.55, then the treatment will be discontinued.
b. *Citrate accumulation*: With the 6 hour citrate infusion, substantive citrate accumulation will not occur and therefore patient serum iCa will not decrease below 0.95 mmol/L as a result of citrate accumulation. If patient's serum iCa drops below 0.95 mmol/L, the patient will receive 1 gram of calcium chloride bolus and ionized calcium rechecked in 1 hour.
c. *Hypernatremia:* Acid citrate dextrose is a hypertonic solution with sodium concentration of 220 mEq/L. If the sodium load is not removed with UF then hypernatremia may ensue. To avoid sodium load to the patient, the added sodium will be removed on the additional dialyzer filter during the ultrafiltration process. The patient's sodium balance will be kept within 50 mEq of net sodium balance. That will avoid a rise in sodium concentration of more than 2 mEq/L even in patients with small body size secondary to extracorporeal therapy.

Human Experience
In all clinical studies to date no serious alkalotic, citrate accumulation or hypernatremia events were reported as related to SCD therapy even though SCD therapy with CRRT-RCA was performed for up to 7 days.

SCD and RRT Device Mechanical Risks
The risks of this device system (RRT/SCD) are similar to risks associated with RRT treatments, and include clotting of the circuit (noted above), air entry into the circuit, catheter or blood tubing kinking or disconnection, and temperature dysregulation.

Risk Minimization
The RRT devices and associated blood perfusion sets have been designed to identify these problems during treatment with alarm systems and to mitigate air embolism to the patient with air bubble traps. All RRT devices and extracorporeal blood tubing sets used in this study are FDA cleared. Studies have been done to verify that the SCD may be used safely

with the RRT devices to be used in this study. The SCD will be observed and changed if any evidence of leakage, cracks or any physical defects is detected.

Human Experience

No CRRT or RRT device, dialysis instrument or SCD device defects have been reported in any of the prior SCD studies.

Additional Safety Procedures to Minimize Risk

The additional procedures minimizing risk in this study include carefully defined inclusion and exclusion criteria, study termination triggers, study monitoring procedures and device monitoring for clotting, leakage, and membrane breakage.

Hypotension

Advanced heart failure patients who are being considered for left ventricular assist device therapy are at risk for hemodynamic compromise from hypotension. The incremental risk of hypotension from RRT and SCD is small, as there will be no net volume removal

Risk Minimization

The UF rate for hemofiltration during SCD has been designed to remove only the sodium and free water load derived from the acid citrate dextrose-A (ACD-A) infusion rate, so that *no net volume will be removed during SCD treatment*. If patients develop hypotension requiring clinical intervention, vasopressor support as per standard medical practice will be utilized. If hypotension occurs in the setting of unexpected filter clotting event, blood transfusion will be utilized with acknowledged safety outcomes identified in the informed consent.

3.3 Potential Benefits

The potential benefits of the SCD include improvement in renal function sufficient to render the patient eligible for LVAD implantation, and improvement of heart function.

3.4 Justification for the Investigation

Patients enrolled in this study will have end stage heart failure and worsening renal function as a result of cardiorenal syndrome or severe right ventricular failure (RVF). Individuals with this degree of heart failure are at very high risk of death in days to weeks, if they cannot be treated with advanced heart failure therapies (i.e., durable ventricular assist device implantation or transplantation). However, these patients’ kidney dysfunction precludes these advanced therapies. SCD therapy offers the possibility of improving kidney function and thereby allowing urgent and life-saving implantation of a LVAD to individuals who have no other viable options to prolong their life.

4 OBJECTIVES

4.1 Primary Objective

To evaluate the safety of 6-hour daily SCD treatment with regional citrate anticoagulation (RCA) in intensive care unit (ICU) patients with NYHA Stage IIIB or IV acute on chronic systolic heart failure (HF), and worsening renal failure (WRF) due to cardiorenal syndrome (CRS) or severe right ventricular failure (RVF), when used as a bridge to durable left ventricular assist device (LVAD) implantation.

4.2 Secondary Objectives

To evaluate the effect of 6-hour daily SCD treatments with RCA to improve renal function while maintaining or reducing pulmonary capillary wedge pressure (PCWP) in ICU patients with NYHA Stage IIIB or IV acute on chronic systolic HF and WRF due to CRS, when used as a bridge to durable LVAD implantation.

5 STUDY OUTCOMES

5.1 Safety Outcomes

1. Need for continuous IV vasopressor support > 5 norepinephrine equivalents > 4 hours following termination of daily SCD therapy session, with norepinephrine equivalents defined as:

   Norepinephrine equivalents: [norepinephrine (mcg/min)] + [epinephrine (mcg/min)] + [dopamine (mcg/kg/min)/2] + [phenylephrine (mcg/min)/10] + [vasopressin (units/min)/0.002]

   (*Note that inodilators (i.e., dobutamine and milrinone) are not components of this measure.)*
2. MI
3. Death
4. Adverse events due to:
   a. SCD
   b. Central venous catheter
   c. RRT device

Surviving patients who reach a safety outcome will be removed from the study.

5.2 Efficacy Outcomes

5.2.1 Principal Outcomes

Among patients with WRF, the percentage of subjects with reversal of WRF (≥ 0.5 mg/dL reduction of serum creatinine from level at study entry), and achieving an eGFR > 30 ml/min/1.73 m^2 and PCW* at or below level at study entry at termination of SCD therapy.

Among subjects with severe right ventricular failure, the percentage of subjects who no longer have severe right ventricular failure, as evidenced by absence of 3 or more of the following 4 indicators of right ventricular failure:

1) CVP > 16 mmHg
2) CVP/PCWP > 0.65
3) RVSWI < 300 mmHg * ml/m
4) PAPi < 2

5.2.2 Main Secondary Outcome

Percentage of subjects receiving a left ventricular assist device

5.2.3 Secondary Outcomes

(Unless otherwise noted, each assessed as change from onset of intervention to 3 and 6 days after initiation of SCD treatments and from onset of intervention to end of SCD support prior to LVAD implantation)

Laboratory Outcomes

1. Change in 24 hour urine volume and sodium, creatinine, urea, creatinine clearance and urea clearance
2. Change in PCWP*
3. Change in serum sodium, potassium, dissolved CO2, BUN, creatinine
4. Change in right ventricular fractional area change, TAPSE and right ventricular global longitudinal strain

Clinical Outcomes

1. Percentage of subjects with ≥ 0.5 mg/dL reduction of serum creatinine from level at study entry, and PCWP* ≤ 18 mmHg
2. Percentage of subjects receiving a left ventricular assist device with serum creatinine ≥ 0.5 mg/dL below level at study entry 30 days following discontinuation of SCD

5.2.4 Exploratory Outcomes

(Unless otherwise noted, each assessed as change from onset of intervention to 3 and 6 days after initiation of SCD treatments and from onset of intervention to end of SCD support prior to LVAD implantation)

1. Change in cystatin-C, BNP, hs-tnT, IL-6, TNF-a, IL-1β, hs-CRP, IL-10, norepinephrine, renin, aldosterone, neopterin, osteopontin, sST2.
2. Change in circulating leukocyte phenotypes (neutrophils, monocytes) with phenotypic cell sorting
3. Characterization of macrophage subpopulations within the left ventricular myocardium obtained at the time of LVAD implantation****

* If PCWP cannot be obtained, PADP will be used in its place. When utilizing PADP in place of PCWP for change measures, comparisons will be made to baseline PADP.
** eGFR calculated using the 4-variable MDRD equation
*** Recognizing that this is not a steady state creatinine
**** This will be obtained from the LV tissue removed for placement of the LVAD inflow cannula as part of usual clinical care

6 STUDY DESIGN

Phase 2, single arm, open label, uncontrolled, before-after, pilot study.

7 SUBJECT POPULATION

This is a single center study that will be conducted at the University of Michigan (Ann Arbor, Michigan). Ten (10) adult patients in an ICU with NYHA class IIIB or IV chronic systolic heart failure will be enrolled and treated.

7.1 Inclusion Criteria

Subjects in whom the subject meet all of the following criteria are candidates for this study:

1) Primary hospitalization for acute decompensated chronic systolic heart failure
2) Potential LVAD candidate with:
    a) Left ventricular ejection fraction ≤25% (for potential destination therapy) or ≤ 35% (for potential bridge to transplantation) as confirmed by baseline imaging procedure
    b) NYHA class IIIB or IV chronic (≤ 90 days) systolic heart failure, with failure to respond to optimal medical therapy (beta blocker, ACE inhibitor or ARB or valsartan/sacubitril, aldosterone antagonist, unless not tolerated or contraindicated, and loop diuretic, as needed) for 45 of the last 60 days
    c) Known previous peak exercise oxygen consumption < 14 mL/Kg/min or if unable to exercise, dependent on an intra-aortic balloon pump, short-term mechanical circulatory support device or intravenous inotropes unless inotropes contraindicated for clinical reasons (e.g., ventricular arrhythmias)
3) Baseline eGFR** ≥ 40 ml/min/1.73 m2 (baseline defined as the highest known eGFR within 90 days of study enrollment)
4) At least one of the following two criteria:
    a) Severe right ventricular failure (RVF), defined as meeting at least 2 of the following 4 criteria
        i) Central venous pressure > 16 mmHg
        ii) Central venous pressure/Pulmonary wedge pressure >0.65
        iii) Right ventricular stroke work index < 300 mmHg * ml/m^2
        iv) Pulmonary artery pulsatility index (PAPi) < 2,
    b) Worsening renal failure (WRF), defined for the purposes of this study as
        i) Increase serum creatinine ≥ 0.5 mg/dL from baseline (baseline defined as the lowest known serum creatinine within 90 days of study enrollment)

        AND

        ii) eGFR** ≤ 30 ml/min/1.73 m2 based on serum creatinine at enrollment***

AND

iii) Cardiorenal syndrome is the most likely explanation for WRF

AND

iv) Intolerant or inadequately responsive to standard of care diuretic therapy

5) PA catheter in place at the time of enrollment
6) PCW ≥ 20 mmHg
7) Age ≥ 21and ≤ 75 years

** eGFR calculated using the 4-variable MDRD equation
*** Recognizing that this is not a steady state creatinine

7.2 Exclusion Criteria

All subjects in whom the subject meets any of the following criteria will be excluded from the study:

1. Any clear contraindication to LVAD therapy that is unlikely to resolve with improvement in renal function and volume status
2. Prior sensitivity to dialysis device components
3. Bacteremia
4. Temperature ≥ 101.5 F or WBC ≥ 10,000 K/uL or any patient with suspected systemic infection.
5. Active malignancy requiring chemotherapy, biological therapy or radiation therapy
6. The use of intravenous iodinated contrast agent within the prior 72 hours or the anticipated use of such an agent during SCD therapy
7. Need for intravenous vasopressor (i.e., phenylephrine, vasopressin), intravenous vasoconstricting inotrope (i.e., norepinephrine or epinephrine) or dopamine > 3 mcg/kg/min. *(Note: use of vasodilating inotropes [i.e., dobutamine and milrinone] or dopamine at ≤ 3 mcg/kg/min will not preclude study inclusion)*
8. Persistent SBP < 80 mmHg
9. WBC < 4000 K/uL
10. Platelets < 100,000K/uL
11. Serum creatinine > 4 mg/dL or receiving dialysis / CRRT
12. Acute coronary syndrome within the past month
13. Women who are pregnant, breastfeeding a child, or trying to become pregnant
14. Subject not able to sign informed consent, unless they have a legally authorized representative (LAR)
15. Concurrent enrollment in another interventional clinical trial. Patients enrolled in clinical studies where only measurements and/or samples are taken (i.e., no test device or test drug used) are allowed to participate
16. Use of any other investigational drug or device within the previous 30 days

8 STUDY DURATION

8.1 Study Duration

Subjects will be followed until 30 days after their last treatment with the SCD.

9 STUDY PROCEDURES

Subjects who elect to participate in this study will complete the study as outlined below. All tests and measurements should be obtained in accordance with the procedures specified in this protocol. If it is not possible to perform a measurement or examination, the reason for not performing the test or measurement should be documented on the source documents.

9.1 Recruitment and Screening

Candidates for the trial will be identified from the investigator's clinic practice or may be referred by from other clinicians. All screening examination procedures will be performed by the investigator or trained personnel working under the investigator's supervision.

9.1.1 Screening (to be obtained ≤ 2 days prior to enrollment)

After informed consent is obtained, potential candidates will undergo a complete examination to determine their eligibility for study participation. Demographics and a complete history, medical history and current cardiovascular medications will be obtained. Please refer to the study schedule of events table in Section 9.4.5 for specific instructions on how to perform each listed study activity.

The complete screening examination and subject histories will include, where medically possible:

- Demographics (date of birth, gender, ethnicity)
- Medical history
- NYHA Class, INTERMACS Patient Profile
- Current cardiovascular medications
- Vital signs and physical assessment
- Clinical laboratory tests, including complete blood count with differential and platelets, comprehensive metabolic panel, BNP, uric acid and total cholesterol
- 24 hour urine volume, sodium, creatinine, urea, protein, creatinine clearance and urea clearance
- Urinalysis
- eGFR based on 4 variable MDRD equation
- Determination of PCWP and PADP by pulmonary artery catheter

Patients who meet all enrollment criteria and who, after receiving appropriate study information, provide their informed consent (as evidenced by a signed informed consent document), will be enrolled in the study and have the following procedure performed:

- Surface echocardiogram for assessment of left ventricular ejection fraction (LVEF), left ventricular internal diastolic diameter (LVIDd), LV global longitudinal strain (LVGLS), right ventricular longitudinal strain (RVGLS), right ventricular fractional area change (RVFAC), and tricuspid annular plane systolic excursion (TAPSE)

Patients that do not meet study criteria-will not be enrolled into the study. These patients will not be counted in the overall study enrollment numbers, but will be listed on the screening log.

9.2 Concomitant Medication

Subjects are permitted to take prescribed medications only. Chronic medications and antibiotics will not be given during the 6-hour SCD therapy sessions but rather will be given following SCD therapy consistent with their appropriate dosing schedule.

9.3 SCD Therapy (Days 1-6)

Subjects will be placed on SCD for planned 6 hours of daily therapy for up to 6 consecutive days during which time a decision will be made as to the subject’s candidacy for LVAD implantation. If a subject is approved for LVAD implantation, this will be done as soon as feasible.

Select nursing personnel at the University of Michigan dialysis unit will be responsible for administering SCD treatments under the supervision and guidance of the clinical nephrology investigators.
The SCD treatment will be delivered using a two-cartridge system using a type of dialysis equipment commonly used for conventional hemodialysis therapy. The SCD cartridge will be added immediately post-hemofilter to the circuit of a standard hemodialysis system, and treatment will be delivered for 6 hours. Blood exchange will occur using a dialysis catheter.

9.3.1 Description of SCD System

The SCD system is comprised of an SCD cartridge and an SCD circuit. The SCD cartridge is made up of polysulfone hollow fibers housed in a polycarbonate casing. The SCD circuit consists of blood lines, and anticoagulation infusion systems and support equipment (Figure 5).

9.3.2 SCD System Perfusion Initiation Protocol

The SCD will be connected to the priming tubing of a standard hemodialysis circuit through connection tubing. The tubing will connect the extracapillary space (ECS) of the SCD to the priming tubing. Aseptic technique must be used throughout the entire integration procedure. Once the lines are primed and the SCD is in place, the pump system will be maintained in bypass mode until ready to be used for the study.

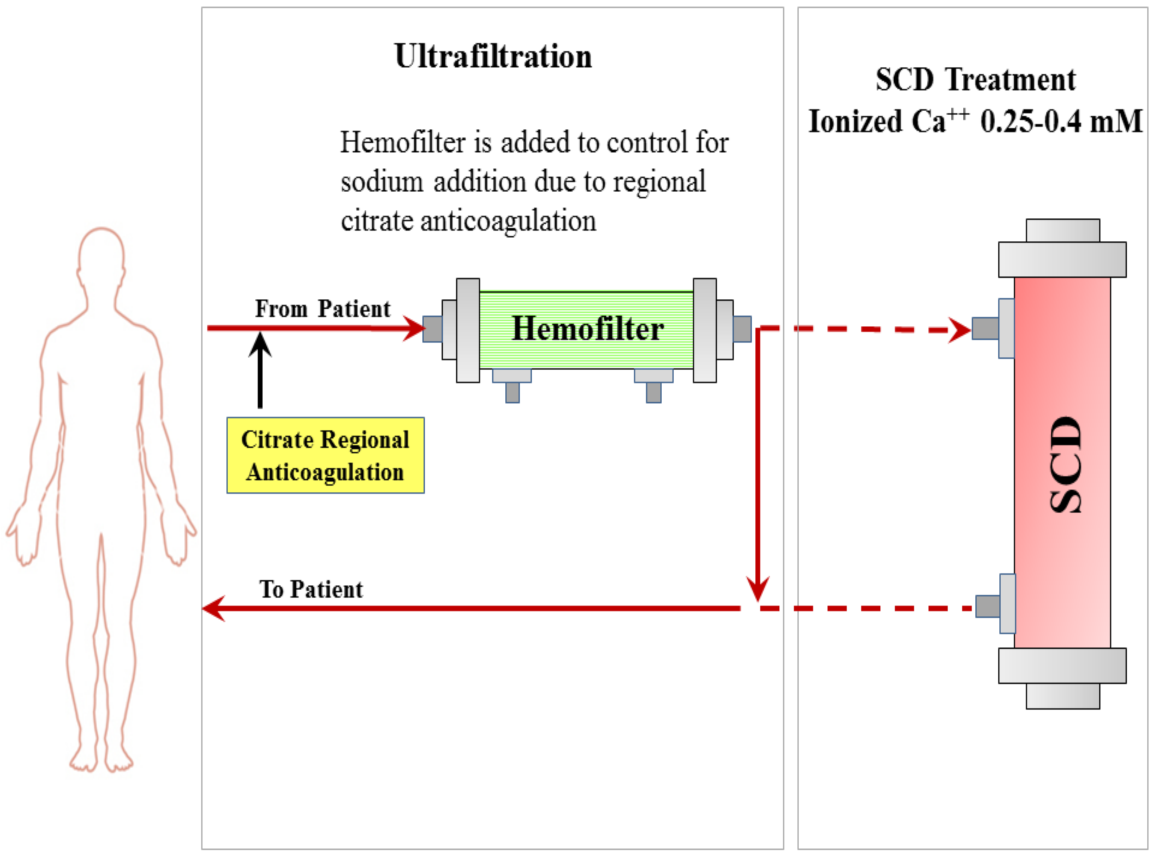

Figure 5. SCD Circuit

9.3.3 Citrate Anticoagulation Therapy

Citrate acts as an anticoagulation agent by binding with calcium. The bound calcium is then unavailable to trigger clotting factors. A fixed blood flow to citrate flow ratio will be used to ensure strong anticoagulant activity throughout the entire circuit.
The citrate enters the patient with the venous blood return and is metabolized in the liver. Systemic ionized calcium levels will be monitored every hour during each 6-hour treatment session.

9.3.4 SCD Prescription Setting

Blood flow rate will range from 60 ml/hr to 150 ml/hr. Hypertonic ACDA citrate will be administered into the circuit pre-hemofilter at 120 ml/hr to 250 ml/hr. Subjects will be administered a calculated volume of 800 ml to 1800 ml of parenteral (in the form of D5W) or enteral free water during SCD treatment to convert the hypertonic sodium load from the ACDA into isotonic sodium gain. Finally, to remove the combined volume of ACDA and free water

intake and the sodium load from the ACDA, isotonic to the patient ultrafiltration during SCD treatment will range from 200 ml/hr to 300ml/hr for 6 hours.

9.3.5 Vascular Access

An acute dialysis catheter will be used for study treatments. The vascular access will be cannulated or accessed only by persons who are trained and experienced in this procedure.

9.3.6 Sampling

Blood samples will be drawn from sampling sites along the extracorporeal blood circuit. Ionized calcium samples will be drawn from extracorporeal blood circuit before citrate infusion site.

9.3.7 Operating SCD Circuit During Normal Treatment Period

We will observe pressure monitors for any consistent, large pressure fluctuations (this could indicate a clotting problem or line occlusion). If the problem persists, dialysis staff will be instructed to call the renal co-investigators. If pressure build up is such that Circuit lines could fail, SCD therapy will be discontinued immediately.

9.3.8 Conclusion of Normal SCD Perfusion

At the conclusion of SCD therapy the patient's blood in the SCD system will be returned to the patient under conventional hemodialysis treatment procedures. Ultrafiltration volume will be recorded and an aliquot sent for measurement of creatinine concentration.

9.3.9 Removal of the SCD

After the conclusion of SCD therapy on Days 1, 3 and 6 the SCD circuit system will be flushed by research personnel with normal saline to clear the SCD cartridge of residual blood. Elution buffer will be infused into the cartridge and all ports capped. Cytometric analysis of the removed SCD will be performed.

9.3.10 Discontinuation of SCD Therapy Due to Adverse Event

If the Principal Investigator or Principal Investigator delegate determines that SCD therapy should be discontinued prior to the scheduled end of treatment, the following procedure should be followed:

a. The dialysate pump system is turned off.
b. A decision to return the blood in the extracorporeal blood circuit to the patient is made.
c. The SCD and its circuit are removed from the dialysis system.

9.3.11 Warnings and Precautions

The following warning and precautionary signs could indicate potential system failures:

a. Changes in patient status should be referred to the clinical investigator.
b. Any leakage of blood from system lines.
c. Any sudden large (>40%), persistent (>2 minutes) change in monitored pressures.
d. Air observed in lines.
e. Development of clots in bubble traps or SCD cartridge extracapillary space.

9.3.12 Required Documentation
The following items need to be recorded:

a. Time of SCD cartridge hook-up.
b. Time of SCD system integration with hemodialysis system.
c. Visual inspection of SCD system upon initiation of perfusion.
d. Calcium levels in blood samples and other parameters as required during Citrate anticoagulation therapy per local protocol.
e. System pump speeds every hour.
f. Adjustments in IV pump infusion rates.
g. Time of cessation of SCD therapy.
h. Any unanticipated occurrences should be recorded in hourly comment sections.

9.3.13 Hemodialysis Cartridges
A new SCD cartridge will be used for each treatment. In the event that either hemodialysis cartridge is not usable for the entire treatment for any reason, a new cartridge will be placed within the perfusion circuit using procedures similar to replacing cartridges during standard hemodialysis.

9.3.14 Disconnecting from the SCD Cartridge
a. All blood in the Study Hemofiltration Circuit will be returned to the Subject (unless contraindicated due to presence of clot, Subject's volume status, etc.).
b. Catheter lumens will be flushed with 10 mL of 0.9% NaCl solution and heparin flush (5,000 units/mL) will be inserted into the ports.

9.4 Study Evaluations

9.4.1 Each Day of SCD Therapy
A study schedule of events table summarizing the follow-up examination schedule and required procedures to be performed at each study visit is provided in Section 9.4.5.
For each day of SCD therapy and the day following VAD therapy, the following blood samples will be obtained each morning:

Blood samples for complete blood count with differential and platelets, comprehensive chemistry panel and BNP.

For each day of SCD therapy, the following procedures will be performed <u>prior</u> to beginning the 6-hour treatment session:

- Temperature, blood pressure, heart rate, respiratory rate (and hourly throughout each 6 hour treatment session)
- Physical examination

- Blood samples for serum ionized calcium will be drawn at baseline and hourly during each 6-hour treatment session.
- eGFR based on 4-variable MDRD equation
- Circuit ionized calcium (and at 2, 4 and 6 hours of each 6 hour treatment session)
- 24 hour urine volume and sodium, creatinine, urea, creatinine clearance and urea clearance
- Urinalysis (Day 6 only)
- Recording of PA, PCW and RA pressures (and again at the end of each 6 hour treatment session)
- Assessment for adverse events

The following procedures will be performed after completing each SCD therapy session

- Physical examination
- CBC with platelets and differential
- Serum sodium, potassium, chloride, CO2, BUN, creatinine, glucose

The following blood samples may be obtained at the discretion of the principal investigator and co-principal investigators just prior to initiation of SCD therapy for each treatment day and 18 (± 6) hours after completion of the last SCD therapy and at the time of LVAD implantation (if ≥ 18 hours after termination of last SCD therapy).

- Blood for cystatin-C, hs-tnT, IL-6, TNF-a, IL-1β, hs-CRP, IL-10, renin, aldosterone, neopterin, osteopontin, sST2
- Blood for circulating leukocyte phenotypes (neutrophils, monocytes) with phenotypic cell sorting

Surface echocardiograms for assessment of LVEF, LVIDd, LVGLS, RVGLS, RVFAC, and TAPSE will be obtained following the 3rd (+ 2days) and 6th (+ 2days) SCD therapy sessions. The echocardiogram obtained following the 6th SCD therapy session may be done up to 2 days after therapy in patients who do not proceed to LVAD.

1. At or before 6 days of SCD therapy, a decision will be reached by the patient's clinicians as to whether or not the subject is eligible for LVAD surgery.
2. SCD therapy will terminate if it is determined that the subject is not a candidate for LVAD therapy, or after 6 days of SCD therapy, whichever comes first.

Other optional diagnostic tests may be performed at the investigator's discretion to further evaluate the subject's outcome or as dictated by the clinical situation. The results of optional diagnostic tests will not be recorded on the CRFs.

All scheduled oral medications and intravenous antibiotics will be given at least three hours prior to SCD therapy or following SCD therapy.

9.4.2 LVAD Implantation

If LVAD implantation occurs we will characterize the macrophage subpopulations within the left ventricular myocardium obtained at the time of LVAD implantation. This will be obtained from the LV tissue removed for placement of the LVAD inflow cannula as part of usual clinical care

9.4.3 Follow-up after LVAD Implantation (7 days and 30 days post implantation (± 1 week))

7 days (±3 days) and 30 days (± 1 week) after LVAD implantation, the following samples will be obtained:

- Blood for complete blood count with differential and platelets, comprehensive chemistry panel and BNP
- Blood for circulating leukocyte phenotypes (neutrophils, monocytes) with phenotypic cell sorting
- Assessment for adverse events considered by the investigator to be related to SCD therapy

9.4.4 Follow up for non LVAD subjects (30 days ± 2 weeks) after last SCD treatment

For patients who do not receive an LVAD, at the completion of the study subject's participation in this study, the following tests will be performed:

- Blood for complete blood count with differential and platelets, comprehensive chemistry panel and BNP
- Blood for cystatin-C, hs-tnT, IL-6, TNF-a, IL-1β, hs-CRP, IL-10, renin, aldosterone, neopterin, osteopontin, sST2 (only for patients who do not receive an LVAD)
- Blood for circulating leukocyte phenotypes (neutrophils, monocytes) with phenotypic cell sorting for adverse events
- Assessment for adverse events considered by the investigator to be related to SCD therapy

9.4.5 Schedule of Events Table

	Screen	Enrollment	Daily while receiving SCD therapy	18 ± 6 hours after completing final SCD therapy	LVAD Implant	Follow-up LVAD Patients 7± 3 days and 30 +/- 7 days) post LVAD Implant	Follow-up non-LVAD patients 30 d (+/_ 2 weeks)after last SCD treatment
Informed Consent	X						
Demographics	X						
Medical Hx/Medication Hx	X		X				
NYHA class, INTERMACS Patient Profile	X				X		
Vital signs: Temp, BP, HR, RR	X		X-hourly during therapy				
Physical Assessment	X		X-before and after SCD therapy				
Urine output (From previous 24 hours) EGFR	X						
Clinical Laboratory Tests- CBC with differential, plts, CMP, BNP	X		X -before SCD therapy)		X	X	X
Sodium, potassium, chloride, CO2, BUN, creatinine, glucose			X -after SCD therapy				
CBC with differential, plts			X -after SCD therapy				
Ultrafiltrate creatinine concentration			X-after SCD therapy				
Uric acid, total cholesterol	X						
Serum ionized calcium			X-every hour				

	Screen	Enrollment	Daily while receiving SCD therapy	18 ± 6 hours after completing final SCD therapy	LVAD Implant	Follow-up LVAD Patients 7± 3 days and 30 +/- 7 days) post LVAD Implant	Follow-up non-LVAD patients 30 d (+/_ 2 weeks)after last SCD treatment
Circuit ionized calcium			X-every 2 hours during therapy				
24° urine volume, sodium, creatinine, urea, creatinine clearance and urea clearance.	X		X				
24° urine protein	X						
Urine Pregnancy Test[1]	X						
Urinalysis	X		X – Day 6 only				
Investigative Laboratory Tests[2]			X-pre-therapy	X	X		X
Investigative Laboratory tests [3]			X-pre-therapy	X	X	X	X
PA catheter measurements	X		X- pre and post therapy				
Surface Echo (LVEF, LVIDd, LVGLS, RVGLS, RVFAC, TAPSE)		X	X-Following 3rd and 6th session only				
LV tissue evaluation					X		
Adverse events evaluation	X		X		X	X	X

[1] For women of child bearing age at screening

[2]- Blood for cystatin-C, hs-tnT, IL-6, TNF-a, IL-1β, hs-CRP, IL-10, renin, aldosterone, neopterin, osteopontin, sST2

[3]- Blood for circulating leukocyte phenotypes (neutrophils, monocytes) with phenotypic cell sorting

Treatment Interruptions-Discontinuation Criteria

9.4.6 Treatment Interruption Criteria

The SCD will be interrupted if the circuit exhibits significant clotting that impairs its functionality.

In the event that an urgent procedure is needed (e.g. CT-Scan, MRI), SCD treatment may be interrupted until completion of such procedures. Thereafter, SCD treatment will be reinitiated to complete the full treatment duration, if logistically possible. These patients will not be removed from the study unless consent is withdrawn.

9.4.7 Treatment Discontinuation Criteria

Subjects may be withdrawn from therapy for a variety of reasons. When therapy is discontinued, the follow-up period will immediately begin and data will continue to be collected per protocol.

Treatment Discontinuation Criteria:

- If it is determined that the subject is not a candidate for LVAD therapy, or after 6 days of SCD therapy, whichever comes first
- LVAD implantation
- Heart transplantation
- The subject no longer wishes to participate in the clinical trial
- The subject experiences adverse events that make it no longer in the subject's best interest to continue the clinical trial in the judgment of the patient or investigator
- RRT or SCD related medical events, such as persistent leukopenia or thrombocytopenia, based upon the PI's clinical assessment
- Hemodynamic intolerance to RRT or SCD therapy
- Concomitant medical conditions that necessitate discontinuation of SCD treatment
- Any other reasons the Principal Investigator deems appropriate for discontinuation from the clinical trial

The Principal Investigator will assign a primary reason for therapy termination utilizing the categories. The reason for a subject's discontinuation on the trial should be documented in the appropriate CRF.

9.4.8 Clinical Trial Termination Criteria

The Sponsor Investigator, Institutional Review Board (IRB), or Food and Drug Administration (FDA) officials reserve the right to terminate the clinical trial for safety or administrative reasons at any time.

Conditions that may warrant discontinuation of the trial may include, but are not limited to, the following:

- Discovery of an unexpected, serious or unacceptable risk to the patients enrolled in the study.
- Decision on the part of the device manufacturer to suspend or discontinue testing, evaluation or development of the study product at any time.

9.5 Device Accountability

All use of the SCD will be under the direct supervision of the principal investigator or his/her designee. The investigational devices will be clearly labeled as investigational use only, and have a clearly marked serial number for each device, which clinical staff will record upon receipt of the device. The receipt date and lot number will be recorded, and package sterility confirmed. The devices will be stored at study nephrologist's locked office, with access limited to the study team.

All records of receipt, use, and disposition of the devices will be maintained by the study team. At the completion of the study, there will be a final reconciliation by study personnel of devices shipped, used, and devices remaining. Any discrepancies noted will be investigated, resolved, and documented prior to return or destruction of unused devices.

10 DATA ANALYSIS AND STATISTICAL CONSIDERATIONS

10.1 Sample Size

This pilot study will enroll 10 patients, which will be sufficient to obtain a preliminary signal of efficacy or lack of efficacy. As this is a pilot study, a sample size calculation will not be performed

10.2 Screening

The screening data for all subjects who are screened, but do not meet eligibility criteria, will not be analyzed or tabulated nor collected on the CRFs. The reason for screen failure will be captured on the CRFs.

10.3 Subject Characteristics

The number of subjects included in the safety and/or effectiveness evaluations, subjects completing the study, and the reasons for any withdrawals will be tabulated by counts and percents. Continuous demographic data will be summarized using descriptive statistics. Categorical demographic data will be summarized using counts and percents. Abnormal medical histories, ocular histories, and prior/concurrent medications obtained on the screening visit will be presented in data line listings.

10.4 Outcome Criteria

As this is a pilot study with a very small number of planned subjects, there will be no formal statistical testing of study hypotheses. Analyses will include descriptive statistics on baseline subject characteristics, and on primary, secondary and other outcomes.

11 ETHICAL AND REGULATORY CONSIDERATIONS

This study will be conducted in accordance with FDA's Good Clinical Practice regulations.

11.1 Informed Consent

In accordance with the provision of 21 CFR Part 50, each subject will provide written informed consent for participation in this study prior to the use of the investigational device.

The study will be explained to the prospective subject by the investigator or his designee. The nature of the experimental product will be explained together with potential hazards of the surgical procedure, including any possible adverse reactions. The subject will be informed that he/she is free to terminate participation in the study for any reason. One copy of the signed consent form will be retained in the medical record, and one copy will be given to the subject.

11.2 Institutional Review Board

This protocol and the ICF will be approved initially and reviewed annually by the University of Michigan Institutional Review Board (IRB). Progress reports will be submitted at the completion of the study or at least once yearly, whichever comes first, to the IRB. Serious adverse events will be reported to the IRB and the FDA in accordance with applicable FDA regulations for serious adverse events.

11.3 Complications and Adverse Events

Complications or adverse events that are observed by the investigator or reported by the subject should be recorded on the CRFs. For all study related adverse events and complications, a description of the event, date first observed, any action taken, and ultimate outcome will be recorded.

For all study related adverse effects, sufficient information will be pursued and/or obtained so as to permit 1) an adequate determination of the outcome of the effect (i.e., whether the effect should be classified as a *serious adverse effect*) and; 2) an assessment of the causal relationship between the adverse effect and the investigational device or, if applicable, the other study treatment or diagnostic product(s).

Adverse effects felt to be associated with the investigational device or, if applicable, other study treatment or diagnostic product(s) will be followed until the effect (or its sequelae) or the abnormal test finding resolves or stabilizes at a level acceptable to the investigator-sponsor.

The investigator will classify all study related reportable adverse events as expected or unexpected, and as either not related to the investigational device, possibly related to the investigational device, probably related to the investigational device, or definitely related to the investigational device. The investigational device is both the hemodialysis system and the SCD device.

Since these subjects are critically ill and hospitalized in the ICU, we will only be collecting adverse events considered to be possibly, probably, or definitely related to the modified hemodialysis system and the SCD device. Adverse events considered to be related to the patient's underlying disease will not be recorded.

Adverse events associated with underlying critical illness are also to be considered expected. Such events include, but are not limited to:

- anemia
- atrial fibrillation
- atrial flutter
- atrial tachycardia
- bacteremia
- cardiogenic shock
- death
- disruption of skin integrity
- hyperglycemia
- hypoglycemia
- hypokalemia
- hyponatremia
- hypotension
- hypothermia
- increased oxygenation requirements
- infection
- lactic acidosis
- leukopenia
- liver failure
- non ST-segment elevation myocardial infarction
- pneumonia
- seizure
- sinus tachycardia
- stroke (ischemic and hemorrhagic)
- ST-segment myocardial infarction
- thrombocytopenia
- ventricular fibrillation
- ventricular tachycardia
- worsening dyspnea
- worsening fluid overload
- worsening kidney failure

Any questions as to the expectedness of an adverse event will be discussed with Sponsor-Investigator.

11.3.1 Serious and Unanticipated Adverse Device Effects
An unanticipated adverse device effect (UADE) is defined as “any serious adverse effect on health or safety or any life-threatening problem or death caused by, or associated with, a device if that effect, problem, or death was not previously identified in nature, severity, or degree of incidence in the investigational plan, or any other unanticipated serious problem associated with a device that relates to the rights, safety or welfare of subjects.”

All UADEs will be reported to the IRB according to local policies and to the FDA according the regulations found in 21 CFR 812.150.

11.3.1.1 Sponsor Investigator Responsibilities
The Sponsor-Investigator will promptly review documented adverse effects and abnormal test findings to determine 1) if the abnormal test finding should be classified as an adverse effect; 2) if there is a reasonable possibility that the adverse effect was caused by the investigational device or, if applicable, other study treatment or diagnostic product(s); and 3) if the adverse effect meets the criteria for a *serious adverse effect*.

In accordance with 21 CFR Part 812.150(a)(1) and (b)(1), the sponsor shall promptly report the results of an evaluation of any serious and unanticipated adverse device effect to FDA, the University of Michigan IRBMED and participating investigators (if any) as soon as possible, but not later than 10 working days after the sponsor first receives notice of the effect. Thereafter, the sponsor shall submit such additional reports concerning the effect as the FDA requests. Complications and non-serious or anticipated adverse events should be documented and tabulated but need not be submitted by the sponsor to the FDA as individual reports.

11.3.1.2 Investigator Responsibilities
All serious and unanticipated adverse events should be reported to the sponsor investigator within 24 hours of first learning of the event. Those that are determined to be serious and unanticipated after sponsor investigator review should also be reported to the IRB as required according to the reporting requirements of the University of Michigan IRBMED.

11.4 Monitoring
To assure adequate protection of the rights of human subjects, per 21 CFR §812.40, 812.43 and 812.46, this study will be monitored by the University of Michigan Institute of Clinical and Health Research (MICHR). Routine monitoring will be scheduled at appropriate intervals, with more frequent visits occurring at the beginning of the study. A site activation visit will take place, followed by routine monitoring visits. Additional visits can be scheduled at the request of the Sponsor-Investigator.

The established monitoring plan will ensure the quality and integrity of the data throughout the study conduct to verify adherence to the protocol, completeness and accuracy of study data and samples collected, dispensing and inventory of the device, and compliance with regulations.

11.5 Independent Data Review and Data and Safety Monitoring Committee

To protect the interests of research subjects and ensure that they are not exposed to undue risk, this trial will be monitored by an independent Data and Safety Monitoring Committee (DSMC). The DSMC will been appointed by the Sponsor-Investigator, and shall have no formal involvement with the subjects or the investigation and function independently of Sponsor-Investigator. The DSMC will be composed of two heart failure cardiologists, independent of the sponsor-investigator and co-investigators.

The DSMC will monitor the progress of the trial with a scheduled meeting or conference call. In addition to reviewing Serious Adverse Events (SAEs), the first DSMC meeting will focus on over all safety of the trial and study device and will make a determination as to whether or not the study should proceed.

The DSMC will also serve as the Clinical Events Committee (CEC), and will adjudicate study related adverse events for clinical accuracy and relatedness to the treatment, and will also ensure a unified evaluation by applying standardized event criteria. The CEC will perform final adjudication of adverse events.

11.6 Source Documents / Case Report Forms

Adequate records will be maintained for the study including subject medical and surgical records, signed ICFs, and device use records. All original source documentation will remain at the investigative site. Study data that are stored at the investigator site in any electronic medical records system, including measurements that are obtained electronically, will be printed and retained in the study files.

All study data will be recorded onto CRFs (electronic or paper) designed for the study. If paper CRFs are used, copies of the CRFs will be retained with the sponsor investigator's study files.

11.7 Deviation from the Protocol

The investigator will not deviate from the protocol without prior IRB and sponsor investigator approval, unless such deviation is necessary to manage a medical emergency. The investigator will notify the IRB and the sponsor investigator of any protocol deviation to protect the life, or physical well-being of a subject in an emergency. Such notice shall be given as soon as possible, but in no event any later than 5 working days after the emergency occurred. All other revisions and/or amendments to the protocol that affect subject treatment, study outcome, or subject safety should be submitted in writing to the IRB for approval prior to implementation. The investigator should maintain a record of all protocol deviations showing the dates of, and the reason for, each protocol deviation.

Changes that affect the scientific soundness of the study or the rights, safety, or welfare of human subjects may also require FDA approval, in addition to IRB approval, prior to implementation. The sponsor investigator will obtain such approvals, if required.

12 REFERENCES

1. Ronco C, Haapio M, House AA, Anavekar N, Bellomo R. Cardiorenal syndrome. *J Am Coll Cardiol*. 2008;52(19):1527-1539. doi:10.1016/j.jacc.2008.07.051.

2. Damman K, Valente MAE, Voors AA, O'Connor CM, Van Veldhuisen DJ, Hillege HL. Renal impairment, worsening renal function, and outcome in patients with heart failure: an updated meta-analysis. *Eur Heart J*. 2014;35(7):455-469. doi:10.1093/eurheartj/eht386.

3. Nohria A, Hasselblad V, Stebbins A, et al. Cardiorenal interactions: insights from the ESCAPE trial. *J Am Coll Cardiol*. 2008;51(13):1268-1274. doi:10.1016/j.jacc.2007.08.072.

4. Mullens W, Abrahams Z, Francis GS, et al. Sodium nitroprusside for advanced low-output heart failure. *J Am Coll Cardiol*. 2008;52(3):200-207. doi:10.1016/j.jacc.2008.02.083.

5. Heywood JT, Fonarow GC, Costanzo MR, et al. High prevalence of renal dysfunction and its impact on outcome in 118,465 patients hospitalized with acute decompensated heart failure: a report from the ADHERE database. *J Card Fail*. 2007;13(6):422-430. doi:10.1016/j.cardfail.2007.03.011.

6. Damman K, Navis G, Voors AA, et al. Worsening renal function and prognosis in heart failure: systematic review and meta-analysis. *J Card Fail*. 2007;13(8):599-608. doi:10.1016/j.cardfail.2007.04.008.

7. Metra M, Davison B, Bettari L, et al. Is worsening renal function an ominous prognostic sign in patients with acute heart failure? The role of congestion and its interaction with renal function. *Circulation: Heart Failure*. 2012;5(1):54-62. doi:10.1161/CIRCHEARTFAILURE.111.963413.

8. Kirklin JK, Naftel DC, Kormos RL, et al. Quantifying the effect of cardiorenal syndrome on mortality after left ventricular assist device implant. *J Heart Lung Transplant*. 2013;32(12):1205-1213. doi:10.1016/j.healun.2013.09.001.

9. Courties G, Moskowitz MA, Nahrendorf M. The innate immune system after ischemic injury: lessons to be learned from the heart and brain. *JAMA Neurol*. 2014;71(2):233-236. doi:10.1001/jamaneurol.2013.5026.

10. Vinten-Johansen J. Involvement of neutrophils in the pathogenesis of lethal myocardial reperfusion injury. *Cardiovasc Res*. 2004;61(3):481-497. doi:10.1016/j.cardiores.2003.10.011.

11. Dirksen MT, Laarman GJ, Simoons ML, Duncker DJGM. Reperfusion injury in humans: a review of clinical trials on reperfusion injury inhibitory strategies. *Cardiovasc Res*. 2007;74(3):343-355. doi:10.1016/j.cardiores.2007.01.014.

12. Swirski FK, Nahrendorf M, Etzrodt M, et al. Identification of splenic reservoir monocytes and their deployment to inflammatory sites. *Science*. 2009;325(5940):612-616. doi:10.1126/science.1175202.

13. Nahrendorf M, Swirski FK, Aikawa E, et al. The healing myocardium sequentially mobilizes two monocyte subsets with divergent and complementary functions. *J Exp Med*. 2007;204(12):3037-3047. doi:10.1084/jem.20070885.

14. Nahrendorf M, Pittet MJ, Swirski FK. Monocytes: protagonists of infarct inflammation and repair after myocardial infarction. *Circulation*. 2010;121(22):2437-2445. doi:10.1161/CIRCULATIONAHA.109.916346.

15. Frantz S, Nahrendorf M. Cardiac macrophages and their role in ischaemic heart disease. *Cardiovasc Res*. 2014;102(2):240-248. doi:10.1093/cvr/cvu025.

16. Leuschner F, Panizzi P, Chico-Calero I, et al. Angiotensin-converting enzyme inhibition prevents the release of monocytes from their splenic reservoir in mice with myocardial infarction. *Circ Res*. 2010;107(11):1364-1373. doi:10.1161/CIRCRESAHA.110.227454.

17. van der Laan AM, Horst Ter EN, Delewi R, et al. Monocyte subset accumulation in the human heart following acute myocardial infarction and the role of the spleen as monocyte reservoir. *Eur Heart J*. 2014;35(6):376-385. doi:10.1093/eurheartj/eht331.

18. Tsujioka H, Imanishi T, Ikejima H, et al. Impact of heterogeneity of human peripheral blood monocyte subsets on myocardial salvage in patients with primary acute myocardial infarction. *J Am Coll Cardiol*. 2009;54(2):130-138. doi:10.1016/j.jacc.2009.04.021.

19. Yndestad A, Damås JK, Oie E, Ueland T, Gullestad L, Aukrust P. Systemic inflammation in heart failure--the whys and wherefores. *Heart Fail Rev*. 2006;11(1):83-92. doi:10.1007/s10741-006-9196-2.

20. Dixon D-L, Griggs KM, Bersten AD, De Pasquale CG. Systemic inflammation and cell activation reflects morbidity in chronic heart failure. *Cytokine*. 2011;56(3):593-599. doi:10.1016/j.cyto.2011.08.029.

21. Conraads VM, Bosmans JM, Schuerwegh AJ, et al. Intracellular monocyte cytokine production and CD 14 expression are up-regulated in severe vs mild chronic heart failure. *J Heart Lung Transplant*. 2005;24(7):854-859. doi:10.1016/j.healun.2004.04.017.

22. Simms MG, Walley KR. Activated macrophages decrease rat cardiac myocyte contractility: importance of ICAM-1-dependent adhesion. *Am J Physiol*. 1999;277(1 Pt 2):H253-H260.

23. Pitt B, Zannad F, Remme WJ, et al. The effect of spironolactone on morbidity and mortality in patients with severe heart failure. Randomized Aldactone Evaluation Study

Investigators. *New England Journal of Medicine*. 1999;341(10):709-717. doi:10.1056/NEJM199909023411001.

24. Usher MG, Duan SZ, Ivaschenko CY, et al. Myeloid mineralocorticoid receptor controls macrophage polarization and cardiovascular hypertrophy and remodeling in mice. *J Clin Invest*. 2010;120(9):3350-3364. doi:10.1172/JCI41080.

25. Ismahil MA, Hamid T, Bansal SS, Patel B, Kingery JR, Prabhu SD. Remodeling of the mononuclear phagocyte network underlies chronic inflammation and disease progression in heart failure: critical importance of the cardiosplenic axis. *Circ Res*. 2014;114(2):266-282. doi:10.1161/CIRCRESAHA.113.301720.

26. Ding F, Song JH, Jung JY, et al. A biomimetic membrane device that modulates the excessive inflammatory response to sepsis. Wanunu M, ed. *PLoS ONE*. 2011;6(4):e18584. doi:10.1371/journal.pone.0018584.

27. Ding F, Yevzlin AS, Xu ZY, et al. The effects of a novel therapeutic device on acute kidney injury outcomes in the intensive care unit: a pilot study. *ASAIO J*. 2011;57(5):426-432. doi:10.1097/MAT.0b013e31820a1494.

28. Tumlin JA, Chawla L, Tolwani AJ, et al. The effect of the selective cytopheretic device on acute kidney injury outcomes in the intensive care unit: a multicenter pilot study. *Semin Dial*. 2013;26(5):616-623. doi:10.1111/sdi.12032.

29. Humes HD, Sobota JT, Ding F, Song JH, RAD Investigator Group. A selective cytopheretic inhibitory device to treat the immunological dysregulation of acute and chronic renal failure. *Blood Purif*. 2010;29(2):183-190. doi:10.1159/000245645.

30. Tumlin JA, Galphin CM, Tolwani AJ, et al. A Multi-Center, Randomized, Controlled, Pivotal Study to Assess the Safety and Efficacy of a Selective Cytopheretic Device in Patients with Acute Kidney Injury. Cravedi P, ed. *PLoS ONE*. 2015;10(8):e0132482. doi:10.1371/journal.pone.0132482.

31. Wrigley BJ, Lip GYH, Shantsila E. The role of monocytes and inflammation in the pathophysiology of heart failure. *European Journal of Heart Failure*. 2011;13(11):1161-1171. doi:10.1093/eurjhf/hfr122.

32. Sabbah HN, Stein PD, Kono T, et al. A canine model of chronic heart failure produced by multiple sequential coronary microembolizations. *Am J Physiol*. 1991;260(4 Pt 2):H1379-H1384.

33. Morita H, Suzuki G, Mishima T, et al. Effects of long-term monotherapy with metoprolol CR/XL on the progression of left ventricular dysfunction and remodeling in dogs with chronic heart failure. *Cardiovasc Drugs Ther*. 2002;16(5):443-449.

34. Rastogi S, Mishra S, Zacà V, et al. Effect of long-term monotherapy with the aldosterone receptor blocker eplerenone on cytoskeletal proteins and matrix metalloproteinases in dogs with heart failure. *Cardiovasc Drugs Ther*. 2007;21(6):415-422. doi:10.1007/s10557-007-6057-8.

35. Simmons EM, Himmelfarb J, Sezer MT, Chertow GM, Mehta RL, Paganini EP, Soroko S, Freedman S, Becker K, Spratt D, Shyr Y, Ikizler TA, et al. Plasma cytokine levels predict mortality in patients with acute renal failure. Kidney Int 2004; 65:1357–1365.

36. Mutunga M, Fulton B, Bullock R, Batchelor A, Gascoigne A, Gillespie JI, Baudouin SV. Circulating endothelial cells in patients with septic shock. Am J Respir Crit Care Med 2001; 163:195–200.

37. Himmelfarb J, McMonagle E, Freedman S, Klenzak J, McMenamin E, Le P, Pupim LB, Ikizler TA, et al. Oxidative stress is increased in critically ill patients with acute renal failure. J Am Soc Nephrol 2004; 15:2449–2456.
